# Outpatient management of acute uncomplicated appendicitis after laparoscopic appendectomy: a randomized controlled trial

**DOI:** 10.1186/s13017-022-00465-5

**Published:** 2022-11-23

**Authors:** Jordi Elvira López, Ricard Sales Mallafré, Erlinda Padilla Zegarra, Luis Carrillo Luna, Joan Ferreres Serafini, Roisin Tully, Robert Memba Ikuga, Rosa Jorba Martin

**Affiliations:** 1 General and Digestive Surgery Department, University Hospital of Tarragona Joan XXIII, Rovira i Virgili University, Tarragona, Spain; 2 Institut d’investigació Sanitaria Pere Virgili (IISPV), 43007 Tarragona, Spain; 3 Anesthesiology Department, University Hospital of Tarragona Joan XXIII, Rovira i Virgili University, Tarragona, Spain; 4 General Surgery Department, St Vincent’s University Hospital, Dublin, Ireland

**Keywords:** Appendicitis, Acute appendicitis, Uncomplicated acute appendicitis, Outpatient management, Laparoscopic appendectomy, ERAS protocol

## Abstract

**Objective:**

To confirm the safety and efficacy of outpatient management of laparoscopic appendectomy, with an enhanced recovery after surgery (ERAS) protocol, in adult patients with uncomplicated acute appendicitis.

**Summary background data:**

Outpatient laparoscopic appendectomy is feasible and secure in selected patients in observational studies. The benefits include reduced length of stay (LOS) and postoperative complications. This is the first randomized controlled trial of outpatient management following ERAS protocol.

**Methods:**

Patients admitted from the emergency department with acute appendicitis were randomized into one of two groups: standard care within the hospital (HG) or the outpatient group (OG). An ERAS protocol was followed for both groups. Patients in the HG were admitted to the surgical ward. Patients in the OG were referred to the day-surgery unit. The primary endpoint was the length of stay.

**Results:**

Ninety-seven patients were included: 49 in the OG and 48 in the HG. LOS was significantly shorter in the OG (mean 8.82 h) than in the HG (mean 43.53 h), *p* < 0.001. There was no difference in readmission rates (*p* = 0.320); we observed only one readmission in the OG. No further emergency consultations or complications were observed. The cost saving was $516.52/patient as a result of the intervention.

**Conclusion:**

Outpatient management of appendectomy is safe and feasible procedure in selected patients. This approach could become the standard of care for patients with uncomplicated appendicitis, showing fewer complications, lower LOS and cost.

*Trial registration*: Registration: www.clinicaltrials.gov (NCT05401188) Clinical Trial ID: NCT05401188

## Backgrounds and aim

Acute appendicitis (AA) is one of the most performed surgical procedures in emergency surgery [1]. The treatment is traditionally with laparoscopic appendectomy (LA) [1]. AA has a hazard rate of 1.17 to 1.9 per 1,000 habitants/year and a lifetime risk of 8.6% for men and 6.7% for women. The most common age range is between 25 and 35 [2]. Open appendectomy was first described by George Thomas Martin in 1887 and then by Charles McBurney in 1889. The first laparoscopic appendectomy (LA) was not performed until 1983 by Kurt Semm [3].

Nechay et al. [4] published a modified enhanced recovery after surgery (ERAS) protocol based on the well-known ERAS program for elective (colorectal) surgery [5]. The choice of modified ERAS protocol components was determined by how well they could be adapted to the emergency setting [4, 6]. The behind publications showed that the management of outpatient appendectomy [7] was safe and feasible. This evidence was validated for patients with uncomplicated AA [8].

The first time an appendectomy was performed as outpatient surgery was in 1994 [9]. However, an increase in complications and hospital readmissions was observed. Since then, many systematic reviews have been published [10, 11]. Data reported in 17 studies (mainly retrospective) suggested that outpatient appendectomy might be feasible. Several observational studies have shown that outpatient surgery is not associated with increased readmissions, increased morbidity rates, or reoperation [9, 11–13]. In 2019, de Wijkerslooth [1] published a systemic review and meta-analysis of comparative studies. However, significant clinical heterogeneity was observed, and therefore, no recommendations could be given. Currently available data [11, 14–16] suggest that in selected patients with uncomplicated AA, patients can be managed via an outpatient route, but there is no good-quality evidence for this. To date, no RCTs have been published.

This is the first RCT of outpatient management of AA following ERAS protocol. The aim of this study is to evaluate the efficacy and safety of this management in patients with uncomplicated AA.

## Methods

### Study design and participants

This is a single-center randomized controlled trial consisting of two treatment groups (NCT05401188). This study was designed in accordance with the Declaration of Helsinki guidelines and approved by the Ethics Committee of Pere Virgili Institute (CEIm 081/2019). Patients were recruited at University Hospital of Tarragona Joan XXIII in Spain from the June 1, 2019, to June 1, 2021.

All patients admitted in the emergency department with AA diagnosis were recruited for the study. Saint Antoine’s criteria were evaluated in all patients [17]. These criteria were the following: leukocytosis lower than 15.000; C reactive protein lower than 30 mg; body mass index lower than 30 kg/m2; appendicular diameter lower than 10 mm; and no radiological sign of complication [17]. The radiological diagnosis could be made by ultrasonography (US) or computed tomography (CT) scan.

Eligible patients were approached, and informed consent was taken. After LA had been performed and a non-complicated AA was confirmed, the patients were randomly assigned to the hospitalization group (HG) or outpatient group (OG).

The inclusion criteria were the following: patients over 18 years old with diagnosis of AA, who met at least 4 out of 5 Saint Antoine criteria, ASA less than or equal to 3, patients who did not live alone at a maximum distance of 30 min from the hospital with an adequate cognitive capacity.

The exclusion criteria were the following: pregnancy or breastfeeding, complicated AA, or a surgical time longer than 90 min.

If the criteria were not met or presenting any exclusion criteria, the patient was excluded from the study and followed the usual clinical management according to our center protocol.

### Anesthetic and surgical protocol

To ensure consistency, an anesthetic and surgical protocol was established for both groups. A modified ERAS protocol [4] was followed in both groups.

During preadmission, the ERAS protocol is not available in the emergency setting. The patient was fasted before surgery. The abdomen was prepared using clippers to remove hair from the incision area. The patient was asked to void the bladder before surgery.

The ERAS protocol for anesthetic management was preoperative evaluation. Intraoperative monitoring included: noninvasive blood pressure, EKG, oxygen saturation, heart rate, Bispectral Index(BIS™) and train of four(TOF™). Pre-oxygenation was performed. Rapid sequence induction was achieved using propofol at a dose of 2 mg/kg, remifentanil in continuous infusion starting at 0.2picograms/kg/min and rocuronium at a dose of 1 mg/kg/min. One minute after induction, intubation was carried out using direct Mackintosh laryngoscopy. Intravenous fluid with balanced crystalloids was started at a rate of 5 ml/kg/h as a replacement for potential losses. Anesthetic status was maintained with Remifentanil TIVA (total intravenous anesthesia) between 0.1–0.2 picograms/kg/min continuous infusion, and propofol at a dose of 10 mg/kg/min in the first 10 min and at 8 mg/kg/min during the next 10 min, and finally between 5 and 7 mg/kg/min for the rest of the surgery according to BIS™. Nausea prophylaxis of dexamethasone 4 mg after induction and Ondansetron 4 mg 20 min prior to completion were completed. Multimodal analgesia was preformed with infiltration at the surgical site prior to incision of 10 ml of 2% lidocaine, with an additional subfascial infiltration before aponeurotic closure with 10 ml of 0.2% of Ropivacaine. Intraoperative analgesia is administered with Dexketoprofen 50 mg and Metamizole 2gr. Reversal of neuromuscular blockade with Suggamadex® is achieved according to TOF at the end of the procedure. Finally, the patient is extubated and transferred to the recovery unit.

For the surgical protocol, the patient is positioned supine. Laparoscopic approach begins with pneumoperitoneum performed through a Hasson’s trocar placed in an umbilical incision after infiltration with local anesthesia. A slow and progressive insufflation is performed. Low-pressure (8–9 mmHg) pneumoperitoneum is established. A 5-mm trocar is inserted in the hypogastric area and a 5/10 mm trocar in the left iliac fossa, after infiltration with local anesthesia. The meso-appendix is dissected either using diathermy or by diving the vessels with endoscopic clips. The closure of the appendix stump is commonly carried out with endoscopic loop. The specimen is retrieved through an endoscopic bag. If it is necessary, the right iliac fossa and the Douglas cul-de-sac are flushed with saline solution. The trocars are removed under direct vision; the residual pneumoperitoneum is eliminated through them. Finally, the closure of umbilical fascia is conducted with long-term monofilament resorbable material and staples are placed in the skin.

Following the postoperative ERAS care protocol, postoperative pain intensity was measured at rest on the visual analog scale (VAS) immediately after the patients regained responsiveness and then after 2, 6, 12 and 24 h following the surgical intervention. Analgesia was provided on demand for patients with pain intensity > 5 cm (VAS). Those who developed postoperative nausea and vomiting (PONV) received antiemetics. No intravenous infusions were given postoperatively to any of the patients.

The patients were discharged home if they had no complications, and they accomplished a ALDRETE score.

A telephone survey was conducted on days 2 and 30 after discharge from hospital. The patients were asked about the presence of pain, the episodes of fever and indigestion, any complications and readmissions.

### Randomization and intervention

Randomization was performed using a computer-generated program. Surgeons on-call were responsible for enrollment and treatment allocation, according to sequentially numbered envelops. Enrollment was unblinded for patients and physicians, due to the type of intervention. To reduce biases, the investigators assessing the outcome did not participate in the follow-up or discharge of patients. All patients received detailed written information about their diagnosis and study treatment plan.

After obtaining the informed consent of the study, the patients were admitted and operated following the same anesthetic technique and the same surgical technique by all the members of the on-call team. Once the patient was operated and an uncomplicated appendicitis was confirmed, the patient was randomized to one of the two branches: the HG or the OG.

Patients in the HG, once the surgical intervention was finished, were transferred to the postoperative recovery unit, and later they were discharged to the usual hospital ward. Patients received adequate intravenous fluid resuscitation, based on their individual hemodynamic parameter and fluid balance, and they received analgesia according to personal requirement. In the hospital ward, the usual patient management protocols were followed until a complete recovery and consequently discharged according to the conventional criteria.

Patients in the OG, once operated, were transferred to the surgery-day unit and were later discharged home if they met the ALDRETE criteria [18], in less than 23 h after the intervention [19] (following the same day surgery criteria). If the patient was operated during the night shift, following the advice of major outpatient surgery [19, 20] where overnight stays are allowed, the patient was admitted to the postanesthetic recovery unit and discharged the next day, always in less than 23 h. In case of being discharged after 23 h or not meeting ALDRETE criteria, it was considered a failure of the outpatient treatment.

### Study endpoints

We consider the length of stay (LOS) the best indicator to confirm the safety and efficacy of outpatient management of laparoscopic appendectomy.

The primary endpoint was the LOS. The LOS was calculated from the time of admission in the surgical area to the time of discharge. The secondary endpoints included the failure of outpatient management, complications, readmission, unplanned hospital appointments within 30 days and cost.

### Data collection

The anthropomorphic, radiologic characteristics and blood samples were collected in the emergency department. Quantitative data were obtained from the blood results and from the appendicular diameter. Categorical data were obtained from radiological features. The postoperative complications were evaluated according to the Clavien-Dindo [21] and Comprehensive Complication Index scores [22]. The day and time of admission to the surgical unit and time of discharge were collected as a time variable. Follow-up after discharge, consultations to the emergency department and its reason were assessed. Outpatient follow-up was carried out up to 6 months. The cost analysis study was also conducted.

Data on each patient were collected in a standard form by the research coordinator ensuring the anonymity of the patients. Data were monitored and included in a database using Access© Microsoft 2013, and the statistical study was performed with Stata® inc. 16 version.

### Sample size and statistical analysis

In the hospital reference population, the median of LOS was 2.75 days (SE 0.42), according to a previous review of cases of AA, admitted to the hospital between 2015 and 2018. The population sample was calculated using Stata®, based on a 5%-α and 10% β-risks. In the sample calculation, at least 92 patients were needed to reach study power. A dropout rate of approximately 30% was assumed, and 28 patients were added to the sample size. Based on this, to detect a reduction in hospital stay which meets the requirements for surgery without admission (contemplated up to a maximum of 23 h, or what is the same as 0.95 days), a total of 120 patients was assigned (a total of 60 patients per group). An analysis was conducted, with the exclusion of patients in whom AA diagnosis was incorrect or in whom no exclusion criteria were met (it was decided before any analysis by the data monitoring committee whose members did not know the treatment assignments).

Differences between groups were analyzed using Fisher exact test for categorical variables and t-test or ANOVA test for quantitative variables. *P*-values < 0.05 were considered significant. If after performing the Shapiro–Wilk test the variable did not follow a normal distribution, the p-values were obtained through a permutation test. Multiple comparison was conducted using Wilcoxon nonparametric test for the categorical variables. Fisher and McNemar tests were used to analyze the results of contingency tables. Valid variables were considered if they presented a maximum of 10% of missing values, choosing a multiple imputation with a matrix of 20 values to solve it. All analyses were performed by a statistician.

## Results

### Selected patients and clinical characteristics

**Fig. 1:**
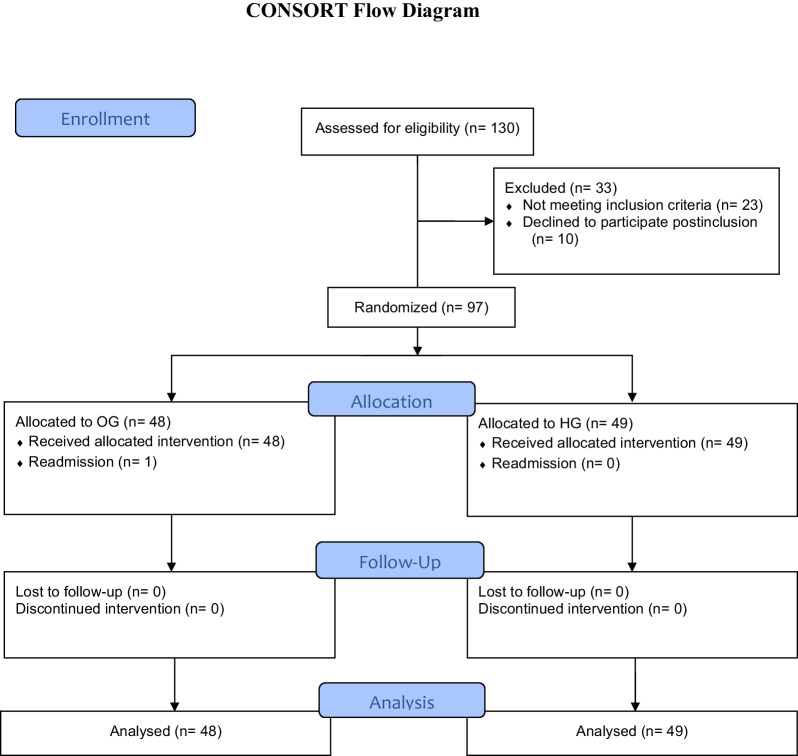
Consort flow diagram—ASI trial. Initial numbers assessed, randomized, followed up, and analyzed with the reason for inclusion

Figure 1 shows the patient selection scheme according to the CONSORT guidelines [23].

A total of 130 patients with AA were recruited to the study; 10 refused to participate. One hundred and twenty patients with AA were initially randomized. After monitorization, 23 patients were excluded from the analysis for not meeting the requirements of the study protocol. Ninety-seven patients were included in the study: 49 in the OG and 48 in the HG. All 97 patients met Saint-Antoine criteria (4 or more items), of which 17 patients (17.55%) had 4 criteria and 80 (82.47%) had all 5 criteria. Table 1 shows the descriptive analysis of the groups. Demographic anthropometric and laboratory data at the time of hospital admission were comparable in the two groups (Table 1).

**Table 1:** Descriptive analysis of the groups: patients characteristics and comorbidity

*N* (%)	OG (*n* = 48)	HG (*n* = 49)	*p*
Sex, *n* (%)			
Male, *n* (%)	29 (60)	21 (39)	0.098
Female, *n* (%)	19 (40)	28 (57)	
Age, mean (sd)	35.30 (14.16)	35.40 ( 14.25)	0.95
BMI, mean (sd)	23.61 (3.12)	26.59 (4.70)	0.0743
Hours of symptoms mean (sd)	36 (0.91)	38 (0.79)	0.8
Fever			1
< 37 °C, *n* (%)	39 (81.63)	41 (84.09)	
37–38 °C, *n* (%)	8 (16.32)	8 (15.91)	
> 38 °C, *n* (%)	1 (2.04)	0	
HTN, *n* (%)	1 (2.08))	1 (2.04)	1
DM, *n* (%)	2 (4.16)	1 (2.04)	0.35
DLP, *n* (%)	1 (2.08)	1 (2.04)	0.65
Cardiac diseases, n (%)	0 (0)	1 (2.04)	0.473
Respiratory diseases, n (%)	1 (2.08)	1 (2.04)	0.725
Another comorbidity, n (%)	0 (0)	0 (0)	–

OG: outpatient group; HG: hospitalization group; yo: years old; BMI: body mass index; HTN: arterial hypertension; DM: diabetes mellitus; DLP: dyslipidemia

### Blood tests inflammatory and biochemical parameters

On the day of admission, the following bloods were obtained. Table 2 shows the descriptive analysis of the groups.

**Table 2:** Descriptive analysis of the groups: blood test analysis

	Global mean (*n* = 97)	OG (*n* = 48)	HG (*n* = 49)	*p*
WBC, mean (sd)	12,252.45 (3283.91)	13,045.1 (516.66)	11,700.9 (421.40)	0.05
Platelets, mean (sd)	229,021.5 (5528.18)	228,816.3 (6923.76)	229,250 (8865.99)	0.97
CRP, mean (sd)	5.09 (0.53)	6.04 (0.81)	4.02 (0.64)	0.06
PT, mean (sd)	1.06 (0.04)	1.10 (0.05)	1.02 (0.04)	0.32
Hemoglobin, mean (sd)	13.86 (0.16)	13.98 (0.20)	13.73 (0.25)	0.45
Creatinine, mean (sd)	0.789 (0.17)	0.789 (0.02)	0.788 (0.02)	0.97
BUN, mean (sd)	28.61 (0.83)	28.90 (1.24)	28.31 (1.10)	0.72

WBC: white blood cell; CRP: C reactive protein; PT: prothrombin time; BUN: Blood urea nitrogen; sd: standard deviation

### Radiological parameters

Preoperative imaging was conducted in all patients (US and/or CT scan). One patient (0.93%) had a suspected intra-abdominal abscess. The mean appendiceal diameter described in the radiology reports was 9.73 mm (SD 2.65 mm). No difference was observed between groups (*p* < 0.05).

### Anesthetic and surgical protocol

The anesthetic variables and the surgical details were individually assessed to confirm that they met the requirements of the study. Overall surgical time was 44.45 min (SD 1.81). In the OG 44.8 min vs 44.05 min in the HG (*p* = 0.833).

## Primary endpoint

The mean LOS was significantly shorter in the OG than in the HG (8.82 h vs 43.53 h, *p* < 0.001). The mean reduction in LOS was − 34.72 h or 0.37 days for the OG and 1.81 days for the HG.

## Secondary endpoints

### Failure of outpatient management

Ninety-nine percent of patients in the OG met the modified ALDRETE criteria and were managed as outpatients; one patient did not tolerate diet due to persistent vomiting and requiring admission.

### Complications, readmissions and unplanned hospital appointments

Table 3 shows the analysis of the groups. In both groups, the Clavien-Dindo 1 complications were poor pain control requiring analgesia. In the HG, there were five patients with Clavien-Dindo 2 complications: 2 bronchospasm and 3 trocar infections requiring oral antibiotics. There were significantly fewer complications in the OG using the CCI (*p* = 0.020). There was no difference between groups in readmissions (*p* = 0.320). No further unplanned hospital appointments were observed in either group.

**Table 3:** Descriptive analysis of complication, readmissions and unplanned hospital appointments

	OG (*n* = 48)	HG (*n* = 49)	*p*
Clavien-Dindo			
0	48 (97.96)	36 (75)	0.018
I	1 (2.04)	8 (16.67)	0.002
II	0 (0)	5 (8.62)	0.3407
III	0 (0)	0 (0)	1
IV	0(0)	0 (0)	1
CCI	0.43 (2.98)	1.57 (3.38)	0.02
Readmission	1 (2.03)	0 (0)	0.32
Unplanned hospital visit	0 (0)	0 (0)	1

CCI: comprehensive complication index

### Cost

The financial department of our hospital provided us with the economic information of the patients. Regarding the cost of the process, because of the fixed cost of the surgical theatre, the healthcare personal cost and the anesthetics cost could be considered equivalent in both groups; the only difference is the time of admission to the hospital ward. The cost saving in the OG was 493.43€ per patient.

## Discussion

AA is one of the most common general surgical emergencies worldwide. The reported life-time risk of appendicitis in the USA is 8.6% in men and 6.7% in women, with an annual incidence of 9.38 per 100.000 persons. In Spain, according to the registry of the Ministry of Health, 44.168 patients were treated for acute AA in 2017 [24].

The severity of clinical classification of AA is based on preoperative assessment. During the WSES in 2015 [1], a group of AA experts discussed many current aspects ending with a new comprehensive disease grading system. Gomes et al. [25] proposed a new comprehensive grading system of AA. Operative findings and intraoperative grading seem to correlate better than histopathology in terms of morbidity, overall outcomes, and costs. This intraoperative grading can determine the optimal postoperative management according to the grade of the disease and the improvement of the utilization of resources [1].

Different options have been described for the treatment of AA. Some authors have proposed a non-surgical treatment [1, 26]. However, major complications have been reported in the antibiotic-alone treatment group, and a high recurrence rate (22.6%) during the first year of the appendicitis episode [1, 26]. For this reason, the COMA trial concludes that surgery should continue to be the mainstay of treatment for AA [27].

Several systematic reviews of RCTs compared LA with open appendectomy. They reported that LA is often associated with longer operative times and higher operative costs, but leads to less postoperative pain, shorter stay, lower incidence of surgical site infection, earlier return to work and physical activity, and better outcomes. Quality of life scores [1, 28]. Thus, in most hospitals in Western countries, LA has become the preferred approach.

The first experience of ambulatory care in the management of AA was published in 2015 by Lefrancois [17] as a prospective descriptive study. Multivariate analysis was performed to create a predictive score of same-day discharge. It allowed to select patients eligible for ambulatorization with a success rate of 97%. However, this study did not assess the severity of appendicitis based on the intraoperative findings. This type of care needs to be validated on a largest cohort.

Trejo-Avila demonstrated in 2019 that implementation of ERAS for appendectomy is associated with a significantly shorter LOS, allowing for outpatient management. The authors concluded that outpatient LA is safe and feasible with similar morbidity and readmission rates compared to conventional care [29].

Recently, Di Saverio in a 2020 update to the WSES Jerusalem [1] guidelines, and Wijkerslooth in a systematic review, established that outpatient LA for uncomplicated AA is feasible and safe with no difference in morbidity and readmission rates. These results are associated with the potential benefits of earlier recovery after surgery and lower hospital and social costs. However, the quality of the evidence was moderate and the strength of recommendation weak (2B).

To date, only four comparative studies have been published in adult patients, using a prospective protocol of a historical control cohort [9, 12, 17, 30]. In addition, two other non-RCT multicenter studies [9, 31] and only one systematic review [11] with significant heterogeneity were published. As a result of this lack of evidence, we decided to design this randomized clinical trial.

Regarding the definition of outpatient criteria, the definitions used so far for early discharge vary widely. For this reason, in the design of our study we used the discharge criteria described by Viñoles [20], Cosse [10], and the Spanish Ministry of Health [19], in which a hospital stay of less than 23 h was defined as the standard for ambulatory surgery. Although these standards do not include emergency procedures, we consider that an appendectomy in selected patients could be comparable with a laparoscopic cholecystectomy, included in group II of the Davis classification [32].

LOS was our primary endpoint. LOS was significantly lower in the OG, 8.82 h (SD 0.83), while in the HG it was 45.43 h (SD 0.96). Coinciding with the literature, where the mean LOS ranged between 3.1 h and 9.6 h [11, 16, 33].

One readmission was observed in the OG for adynamic intestinal ileus. No readmissions were observed in the HG (p = 0.320). Based on similar studies, we have shown that following an ERAS protocol and outpatient management of uncomplicated AA in adult patients is a safe procedure, with low complications and readmission rates ranging from 0 to 4.6% [9, 12, 17, 30].

We observe a lower percentage of complications especially Clavien-Dindo 1 (all related to the presence of postoperative abdominal pain) in the OG than in the HG. For these remarkable and significant findings, there is no clear clinical or pathophysiological explanation despite having evaluated inflammatory parameters, such as C reactive protein and leukocyte levels, and surgical findings. It would be interesting to carry out other studies to try to explain these findings.

In terms of costs, AA is associated with a considerable financial burden due to its high incidence and the cost of hospitalization. The effective use of resources by minimizing costs and maintaining quality is the goal of health care. In 2009, the estimated cost of hospitalization for patients with AA was estimated to be $1,900 in the USA [7]. In our study, health savings were €493.43($516.52) per patient. In the literature, various prospective studies with same-day surgery reported a median reduction in hospital costs ranging from $323 to $4111 per patient [11, 16, 30].

Assessing the possible limitations of this study, the fact that the study design is not blind can be considered a limitation, but the nature of the interventions performed (OG vs HG) made it clear to patients and physicians which group was assigned treatment. Furthermore, the main variables are objective measures, so they are unlikely to be affected by this fact.

Another possible limitation of the study is that the COVID-19 pandemic occurred during the patient recruitment period, which spanned 2020–2021. During the first stage of the COVID-19 pandemic, there was a significant increase in the rate of complicated appendicitis [34]. In addition, a PCR test was added to the protocol, which could increase the hours of stay. However, outpatient management during the pandemic allowed a greater availability of hospital beds to care for patients with medical conditions. Thus, outpatient appendectomy allows better optimization of resources.

In our study design, we decided to calculate a sample size with a high number of follow-up losses. The reason for increasing the sample was to avoid bias due to the low power of the study at the end of data entry. In the sample calculation, a minimum number of 92 patients was obtained to reach the power of the study. To these 92 patients, it was decided to add 30% more patients (28 patients) to avoid bias due to loss of patients. A total of 120 patients were calculated, including estimated losses. At the end of the study, there were 97 patients and 23 actual losses (compared to the previous estimated 28 lost patients). A total of 97 patients exceed the minimum requirements for study potential (which was a minimum of 92 patients).

Our results agree with those reported in most studies [9, 11, 12, 15–17, 35, 36]. We can conclude that in our experience it is possible to start an ERAS protocol and an outpatient appendectomy program with an experienced team. Although several studies [9, 11, 15] had shown, before ours, a reduction in LOS in patients selected for outpatient treatment, it had not been possible to draw solid recommendations due to the significant clinical and methodological heterogeneity between the different studies. Along the same lines, the international guidelines [1] could not give strong recommendations due to the lack of scientific evidence from randomized clinical trials. This is the first RCT on the subject. We have shown that ambulatory appendectomy with the ERAS protocol is safe in selected patients, due to the improvement in terms of quality of care, clinical and economic benefits.

## Conclusion

The emergency outpatient appendectomy with an ERAS protocol is a safe and feasible procedure in selected patients with non-complicated appendectomy. It can be achieved with low morbidity, few readmissions, high patient satisfaction and cost reduction. This approach will become the standard of care for patients with uncomplicated AA in the future.

## Data Availability

The full de-identified database will be made available for independent analysis on reasonable request to the corresponding author or as an appendix in the publishing journal.
